# Supplementary figures and images for: Systematic analysis of noise reduction properties of coupled and isolated feed-forward loops
Source: PLoS Comput Biol. 2021 Dec 3;17(12):e1009622. doi: 10.1371/journal.pcbi.1009622 (PMC8641863; doi:10.1371/journal.pcbi.1009622)

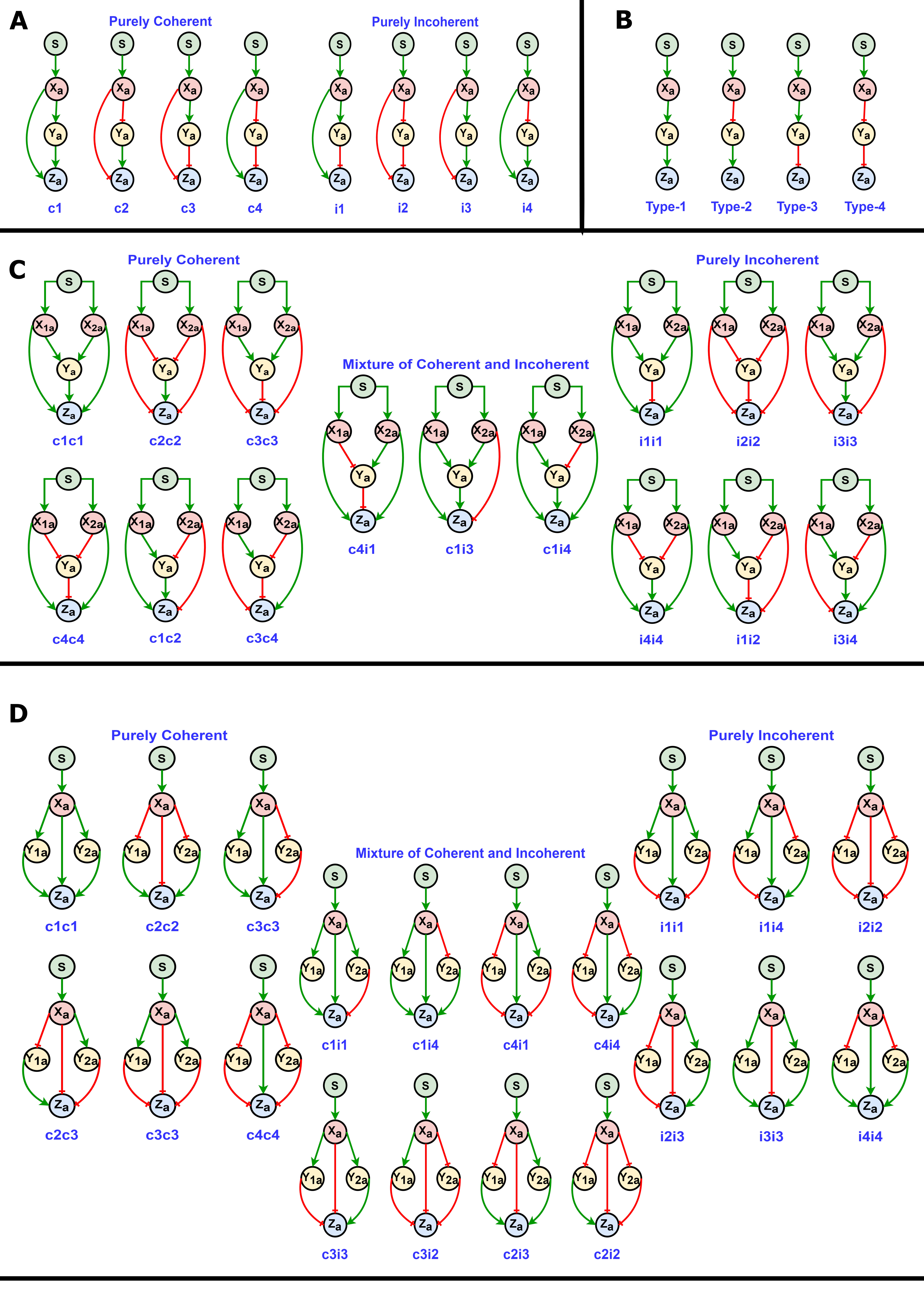

Supplement: S1 Fig — (A) Isolated feed-forward loops: The diagram represents the purely coherent (upper panel) and purely incoherent (lower panel) types of isolated feed-forward loops. Here, the possibilities of AND/OR types of logical gates are taken into consideration. Twelve different kinds of logical connectivity are possible. Coherent and incoherent are represented by the letters ‘c’ and ‘i’ respectively. The number next to the letter ‘c’/ ‘i’ designates the type of model (for example, c1 –coherent type 1, i4 –incoherent type 4 model). Activation and inhibition processes are shown by green and red arrow heads, respectively. The noisy input signal (S) regulates Xa, which in turn influences Ya and the output signal (Za) through the direct and indirect arms of the network. (B) Chain models: The diagram shows all the possible types of chain model. The noisy input signal (S) regulates Xa, which in turn influences Ya, and Ya influences the output signal (Za). (C) Multi-input coupled feed-forward loops: The diagram shows all the multi-input coupled feed-forward loops (minp-FFL). These networks are subcategorized into purely coherent (left panel), mixture of coherent and incoherent (middle panel) and purely incoherent (right panel) types. The input signal (S) jointly activates two nodes, X1a and X2a. These X1a, and X2a influence Ya and Za through direct and indirect regulated arms, where Za represents the output signal. The green arrows represent activation, and the red arrows represent inhibition. Depending on the network architecture, fully AND, fully OR, upper-AND-lower-OR (uAND-lOR) and upper-OR-lower-AND (uOR-lAND) types of logical gates can be considered. (D) Multi-intermediate coupled feed-forward loops: The diagram represents various types of multi-intermediate coupled feed-forward loop (mint-FFL). These networks are subdivided into purely coherent (left panel), mixture of coherent and incoherent (middle panel) and purely incoherent (right panel) types. The noisy input signal (S) r [file pcbi.1009622.s001.tif]

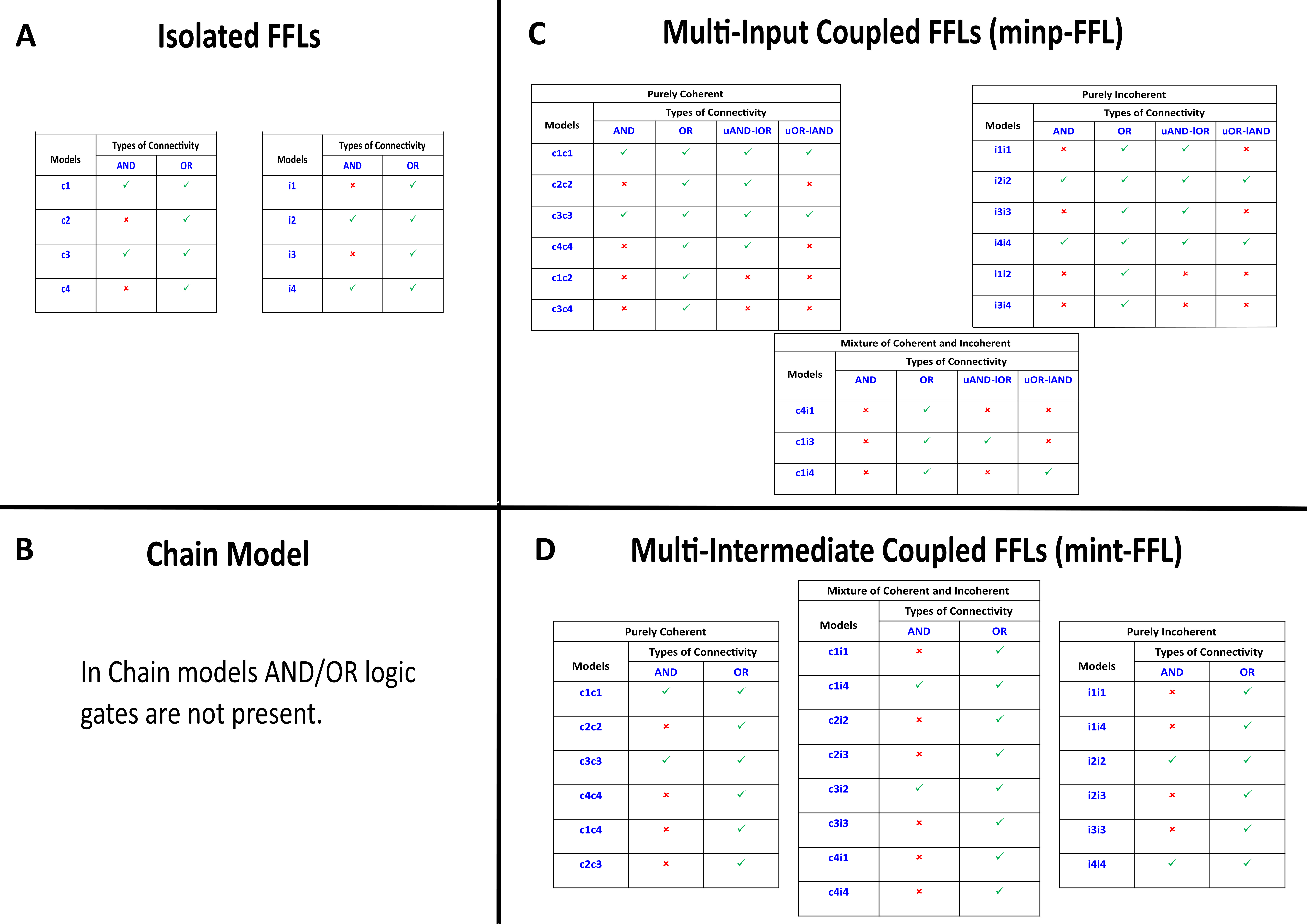

Supplement: S2 Fig — For the investigated network motifs, all the possible logical gates are shown here. Depending on the network architecture, fully AND, fully OR, upper-AND-lower-OR (uAND-lOR) and upper-OR-lower-AND (uOR-lAND) types of logical gates can be created. (A), (B), (C) and (D) show the types of connectivity for 12 isolated feed-forward loops (FFLs), 4 chain models, 33 multi-input coupled feed-forward loops (minp-FFLs), and 26 multi-intermediate coupled feed-forward loops (mint-FFLs), respectively. (TIF) [file pcbi.1009622.s002.tif]

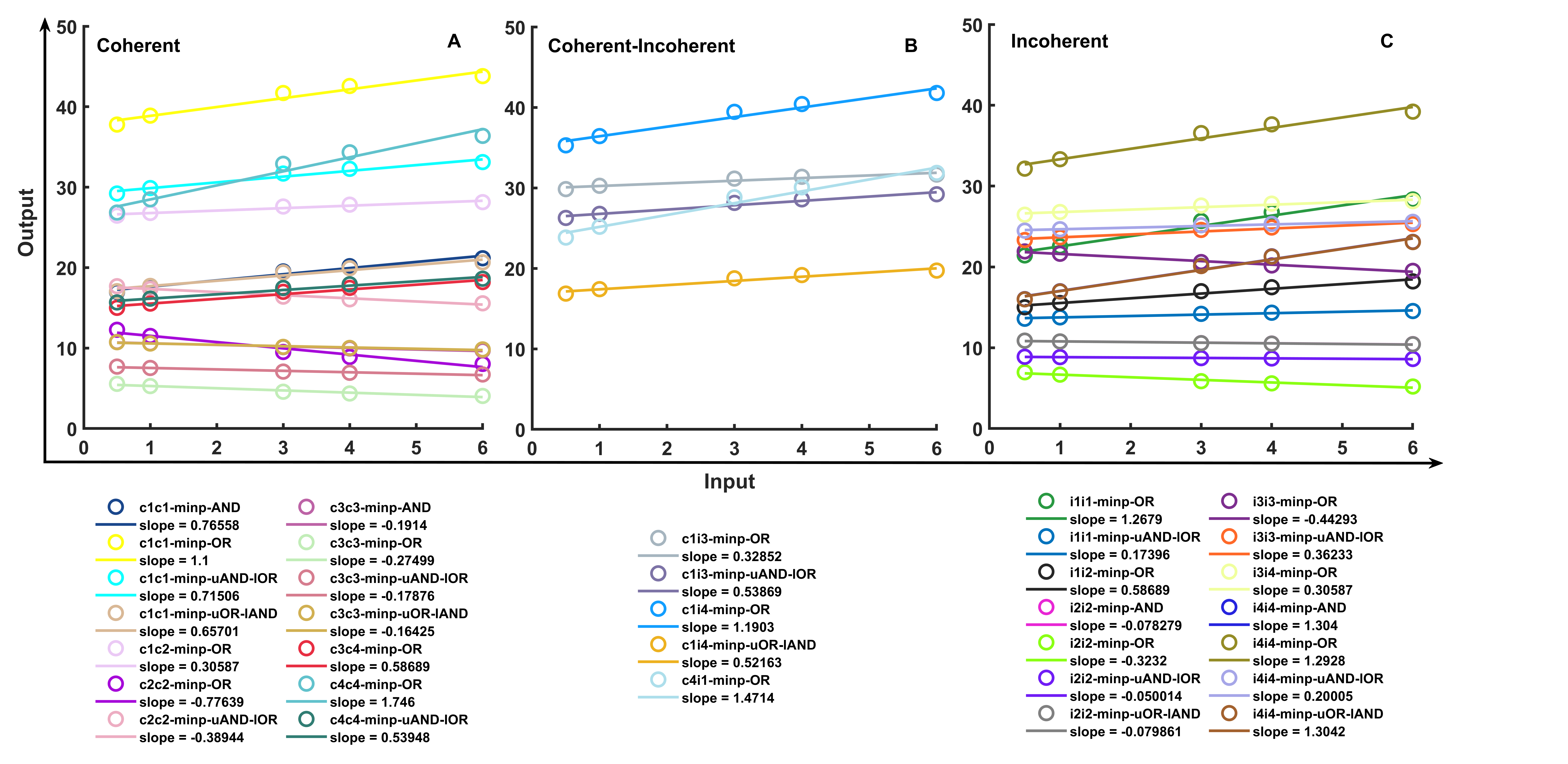

Supplement: S3 Fig — Correlation between input and output signals for multi-input coupled feed-forward loop models, with single step post translation modification of the species under parameter set k1 = k2 = k3 = 1, kp = 10, kpp = 40, ka = 5. The slopes obtained by linear regression for each model are given in the legends. Each model is categorized as purely coherent (A), a mixture of coherent-incoherent (B), and purely incoherent (C) types. Results from all networks presented in S2 Fig (AND, OR, upper-AND-lower-OR (uAND-lOR) and upper-OR-lower-AND (uOR-lAND)) are presented in this plot. Based on the signs of the interactions, both negative and positive correlations can be observed. The input was changed in five steps in each model, as presented in Fig 1F, and the outputs were recorded as in Fig 1G. (TIF) [file pcbi.1009622.s003.tif]

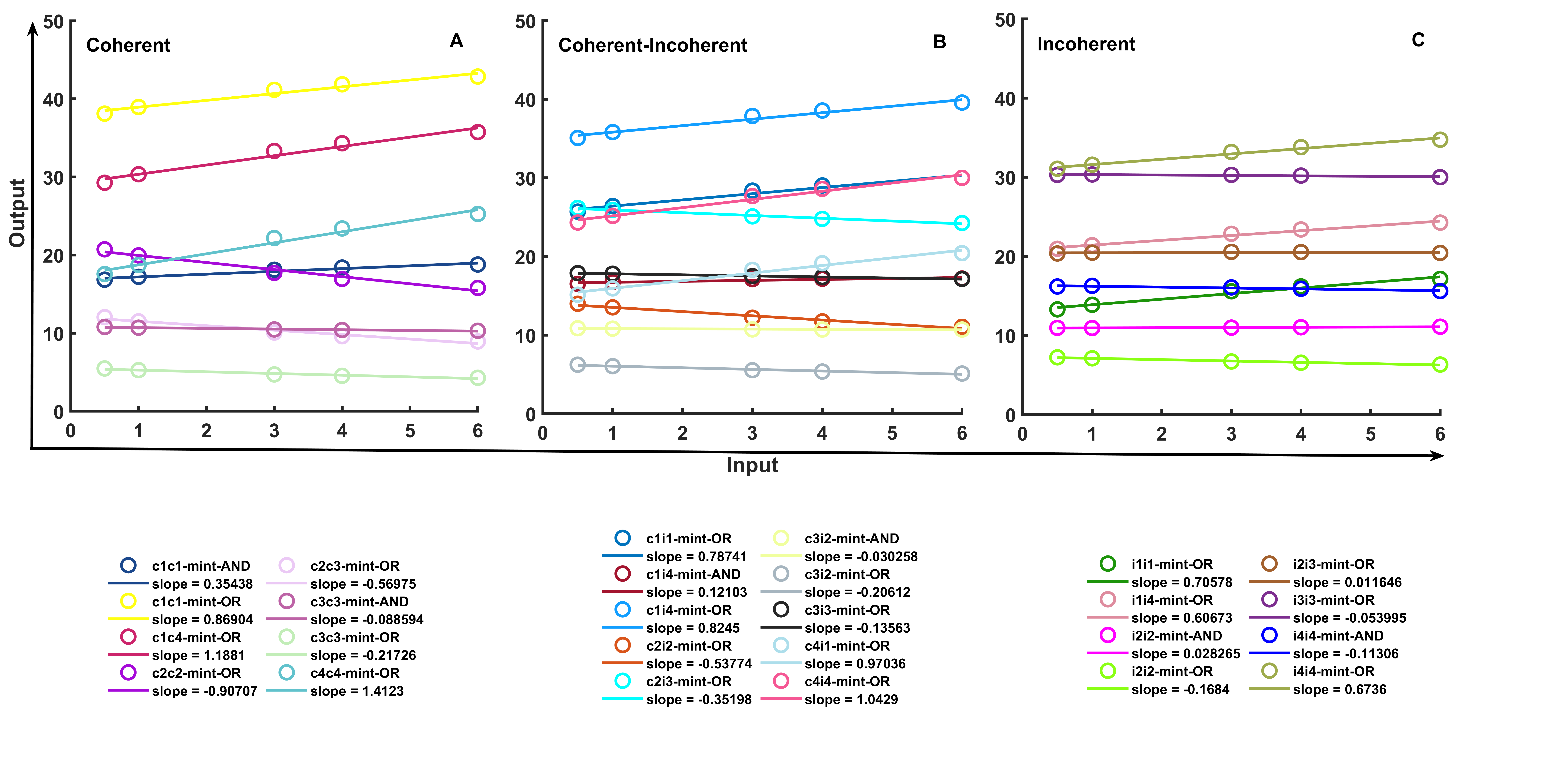

Supplement: S4 Fig — Input-output correlation of steady states in multi-intermediate FFLs for purely coherent (A), mixture of coherent-incoherent (B) and purely incoherent (C) networks are plotted. All possible logical connectivity (AND, OR, upper-AND-lower-OR (uAND-lOR) and upper-OR-lower-AND (uOR-lAND)) have been considered. The input was changed in five steps in each model, as presented in Fig 1F, and the outputs were recorded as in Fig 1G. For the calculation, we have considered single-step post-translation modification of the species under parameter set k1 = k2 = k3 = 1, kp = 10, kpp = 40, ka = 5. (TIF) [file pcbi.1009622.s004.tif]

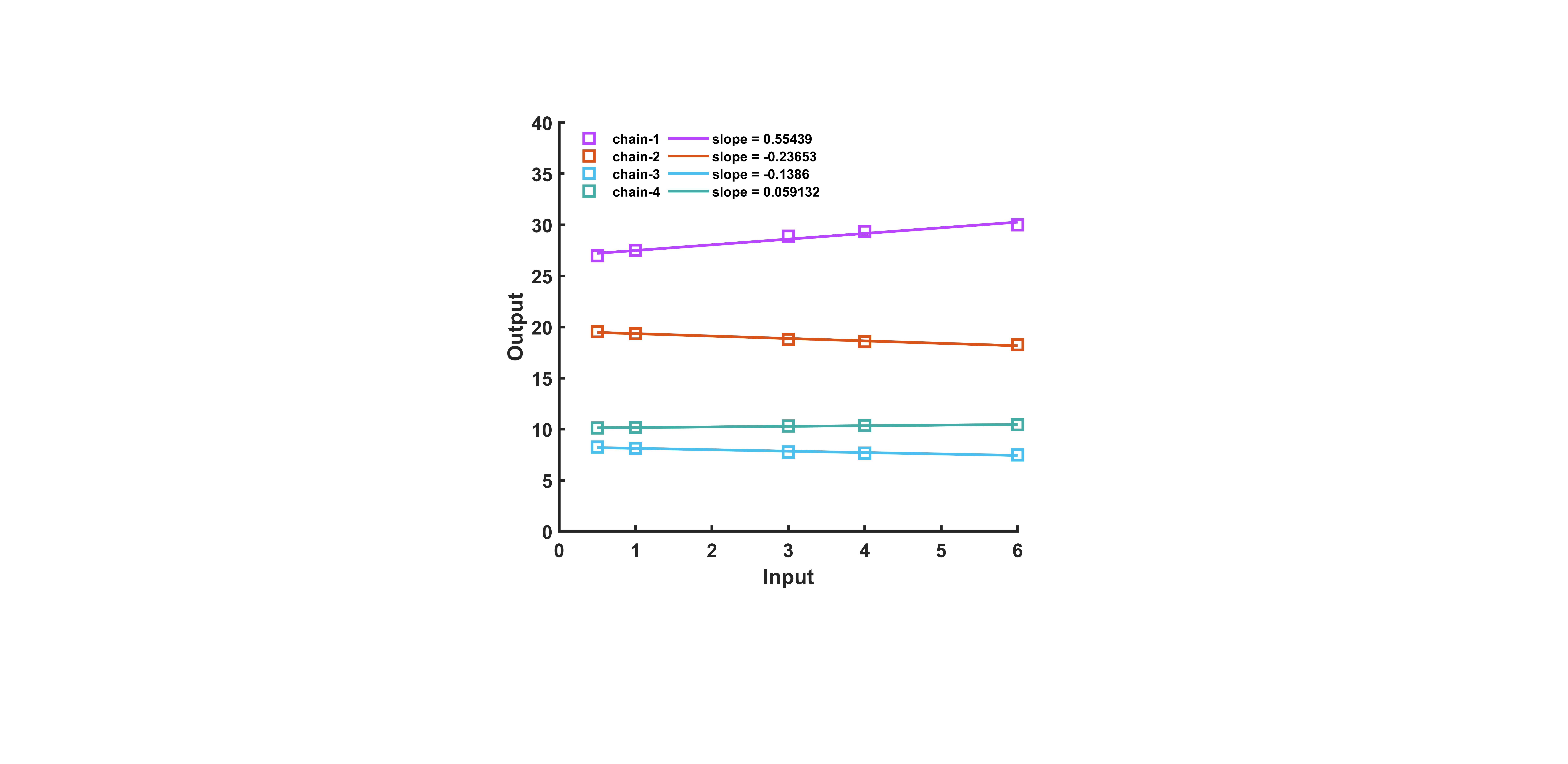

Supplement: S6 Fig — We plot correlation between input and output signals of chain models, considered with single step post translation modification of the models, with parameter set k1 = k2 = k3 = 1, kp = 10, kpp = 40, ka = 5 The caption lists the slope values derived from each model. In each model, the input was modified in five steps as shown in Fig 1F, and the outputs were recorded as shown in Fig 1G. (TIF) [file pcbi.1009622.s006.tif]

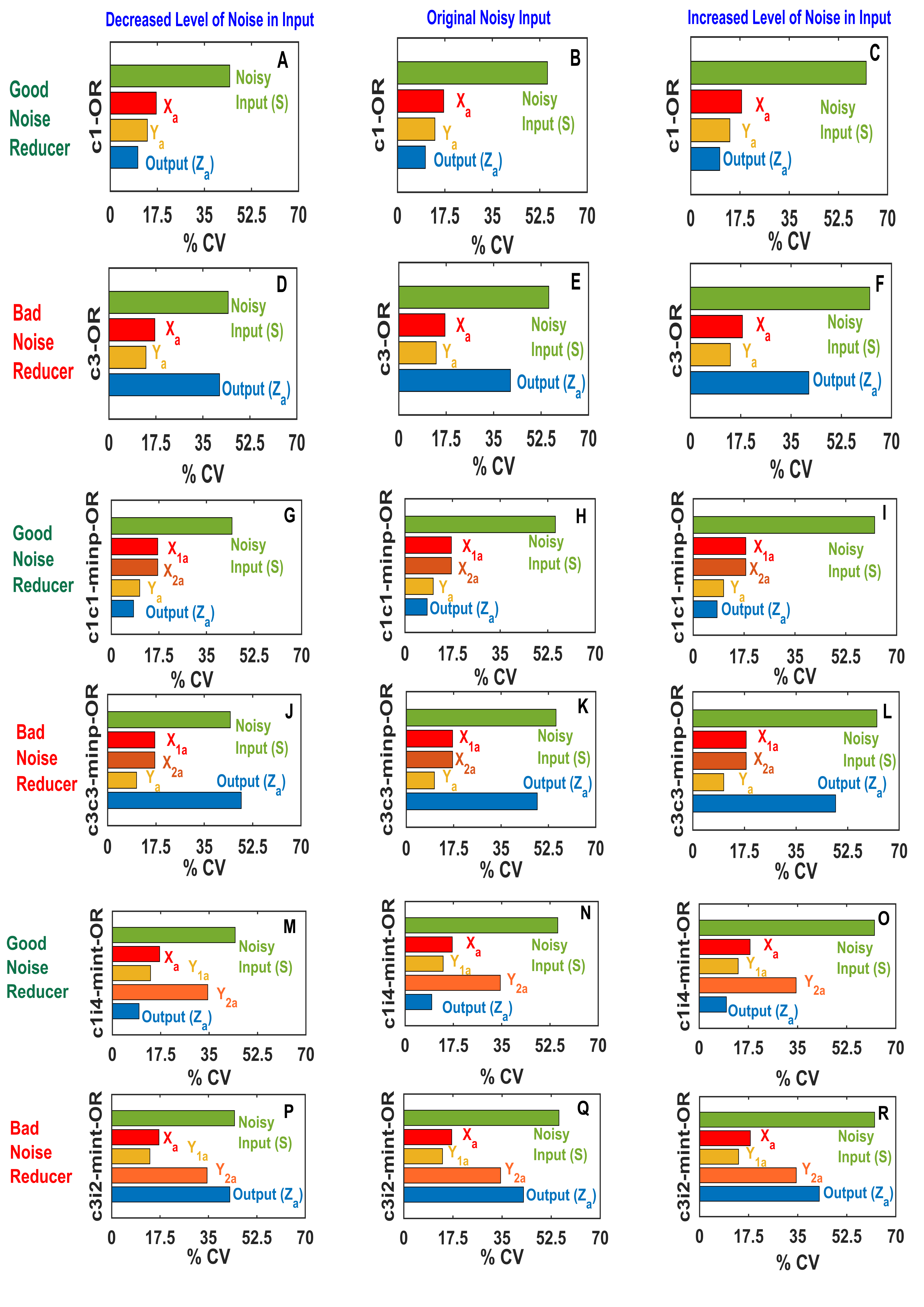

Supplement: S7 Fig — By increasing (right hand column) and decreasing (left hand column) the level of noise in the input (by changing the kinetics of S synthesis, see below in the S1 Text.) and comparing with the original noisy input (middle column), we show how noise propagating through the pathways for c1-OR (A, B, C), c1c1-minp-FFL (G, H, I), c3-OR (D, E, F), c3c3-minp-FFL (J, K, L), c1i4-mint-FFL (M, N, O), and c3i2-mint-FFL (P, Q, R) networks at mean input = 6. The level of noise in the output is largely independent of the degree of noise in the input. We consider single step post translation modification of these networks with parameter set k1 = k2 = k3 = 1, kp = 10, kpp = 40, ka = 5. (TIFF) [file pcbi.1009622.s007.tiff]
