# Supplementary material for: Systematic analysis of noise reduction properties of coupled and isolated feed-forward loops
Source: PLoS Comput Biol. 2021 Dec 3;17(12):e1009622. doi: 10.1371/journal.pcbi.1009622 (PMC8641863; doi:10.1371/journal.pcbi.1009622)
Supplement: S1 Text — (DOCX) [file pcbi.1009622.s008.docx]

**S1 Text. Model generation and parameterization:**

All models have an input signal with super-Poissonian noise (Fano factor > 1). In order to achieve this, we have generated the stochastic input signal S from the deterministic signal A^+^ (which we modified in 5 steps as noted in Fig 1F), by stochastically producing two copies of S at a rate depending on A^+^. The correlated noise in S production leads to super-Poissonian noise (Fano factor = 1.83, Fig 1F). For increased and decreased input noise levels on Figs 7 and S7 we have produced three or only one copy of *S*, respectively. To keep the mean input levels the same, the production rates of these reactions had to be scaled as described below in this section. The noisy input signal S activates the node X (moves into X_a_) at a rate of k_a_. The active form of X (X_a_) influences the intermediary node Y at a rate of k_1_. The influence can be activatory or inhibitory. In the case of activation, Y turns into Y_a_ (active form of Y), while in the inhibition process, Y_a_ turns into Y (inactive form of Y). Both the nodes, X_a_ and Y_a_ , influence the output Z at a rate of k_3_ and k_2_ respectively. Activation will lead to Za (active form of Z) from Z (inactive form of Z), while inhibition will lead to Z from Za. We have accounted for the background reaction for all the species (i.e., X, Y and Z) through phosphorylation and dephosphorylation reactions. The rates for these background phosphorylation and dephosphorylation reactions are k_p_ and k_pp,_ respectively. The background rates are numerically larger than the catalytic rates k_1_, k_2_ and k_3_, but the concentrations of X, Y, Z move between 0 and 60 (AU), thus the maximal propensities of the catalytic reactions are above the propensities of the background reactions. The fixed parameter set is k_1_=k_2_=k_3_=1, k_p_=10, k_pp_=40, k_a_=5. The parameters of all reactions were set in a way to ensure none of the species reaches 0 or maximal 60 (AU) concentrations, to avoid saturation, which would lead to signal loss. By this way, we also ruled out the possibility of noise cancellation due to the saturation of species. To keep the total rates of conversion between forms equal in OR and AND gated models, we introduced a complex formation reaction in AND models. For example, when X1a and X2a are jointly activating Y with a rate constant k1, instead of using 0.5* k1 rate (to match the max rate achievable in an OR model), we have added the complex formation reaction: X1a + X2a {200} <-> {200} C1 (where C1 is the complex formed by X1a and X2a, and at such fast rates, it is always in a steady state balance between the concentration of X1a and X2a). Following this, the C1 complex acts on Y with a rate constant k1: Y + C1 ->{k1} C1 + Ya. In this way, the characteristics of X1a and X2a are carried over to the C1 complex.

As an example, the chemical reaction network of the c1c1-minp-OR FFL model is given here (this is the first example shown as a Kaemika code in S2 Text):

A⁺ -> Ø

A⁺ -> S + S + A⁺

S -> Ø

X1 + K -> K + X1a

X1 + S -> S + X1a

X1a + PP -> PP + X1

X2 + K -> K + X2a

X2 + S -> S + X2a

X2a + PP -> PP + X2

Y + K -> K + Ya

Y + X1a -> X1a + Ya

Y + X2a -> X2a + Ya

Ya + PP -> PP + Y

Z + K -> K + Za

Z + Ya -> Ya + Za

Z + X1a -> X1a + Za

Z + X2a -> X2a + Za

Za + PP -> PP + Z

The second, S producing reaction had to be modified to increase or decrease noise in the input layer *S* (for plots of S7 Fig) in the following way:

*Decrease noise:*

A⁺ -> S + A⁺

with reaction rate doubled from original

*Increase noise:*

A⁺ -> S + S + S + A⁺

with reaction rate 2/3 times the original.
