# Supplementary material for: Systematic analysis of noise reduction properties of coupled and isolated feed-forward loops
Source: PLoS Comput Biol. 2021 Dec 3;17(12):e1009622. doi: 10.1371/journal.pcbi.1009622 (PMC8641863; doi:10.1371/journal.pcbi.1009622)
Supplement: S2 Text — (DOCX) [file pcbi.1009622.s009.docx]

**S2 Text. Examples of Kaemika codes of models**

The Kaemika code of all models are available at <https://github.com/SuchanaChakravarty/Noise-Reduction-Properties-of-Coupled-and-isolated-Feed-Forward-Loops>. The Kaemika software can be downloaded from all APP stores and from <http://lucacardelli.name/>. Here we present a three commented examples to explain how the code works for each model types. Comments in green explain each model building steps and functions of each piece of code.

**Kaemika code for LNA computation of c1c1-minp-OR model under one step post translational modification:4**

^//======================================^

^// c1c1 minp OR model^

^//======================================^

^// Creating step like function^

^//======================================^

^network DSignal(species X⁺ , function f) {^

^number precision = 1000^

^# ->{{precision*pos(f())}} X⁺; X⁺ ->{precision} #^

^}^

^//======================================^

^// Declaration of species^

^//======================================^

^species {A⁺,S,X1,X1a,X2,X2a,Y,Ya,Z,Za,K,PP}^

^//======================================^

^// Initial condition^

^//======================================^

^amount A⁺@ 0M^

^amount X1 @ 60 M^

^amount X1a @ 0 M^

^amount X2 @ 60 M^

^amount X2a @ 0 M^

^amount Y @ 60 M^

^amount Ya @ 0 M^

^amount Z @ 60 M^

^amount Za @ 0 M^

^amount S@ 0M^

^amount K@ 1M^

^amount PP@ 1M^

^//======================================^

^// Parameter value^

^//======================================^

^number k1 = 1^

^number k2 = 1^

^number k3 = 1^

^number kp = 10^

^number ka = 5^

^number kpp = 40^

^//======================================^

^// Input Generation^

^//======================================^

^DSignal(A⁺, fun(){cond(time<40,0.25,cond(time<80,1.5,cond(time<120,3,cond(time<160,2,0.5))))})^

^//======================================^

^// Plot input and output^

^//======================================^

^report S,Za^

^//======================================^

^//The mass-action kinetics reactions:^

^//======================================^

^A⁺ ->{200} S + S + A⁺ // stochastic input generation^

^S ->{200} # // self-degradation^

^X1 + K ->{kp} K + X1a // phosphorylation reaction, i.e. background reaction^

^X1 + S ->{ka} S + X1a // influence of stochastic input on the node of the motif^

^X1a + PP ->{kpp} PP + X1 // dephosphorylation reaction^

^X2 + K ->{kp} K + X2a // phosphorylation reaction, i.e. background reaction^

^X2 + S ->{ka} S + X2a // influence of stochastic input on the node of the motif^

^X2a + PP ->{kpp} PP + X2 // dephosphorylation reaction^

^Y + K ->{kp} K + Ya // phosphorylation reaction, i.e. background reaction^

^Y + X1a ->{k1} X1a + Ya // activation of Y into Ya through OR connectivity^

^Y + X2a ->{k1} X2a + Ya // activation of Y into Ya through OR connectivity^

^Ya + PP ->{kpp} PP + Y // dephosphorylation reaction^

^Z + K ->{kp} K + Za // phosphorylation reaction, i.e. background reaction^

^Z + Ya ->{k2} Ya + Za // activation of Z into Za through OR connectivity^

^Z + X1a ->{k3} X1a + Za // activation of Z into Za through OR connectivity^

^Z + X2a ->{k3} X2a + Za // activation of Z into Za through OR connectivity^

^Za + PP ->{kpp} PP + Z // dephosphorylation reaction^

^//======================================^

^// Total time of simulation^

^//======================================^

^equilibrate for 200^

^//======================================^

^// End^

^//======================================^

**Kaemika code for LNA computation of c1c1-minp-AND model under one step post translational modification:**

^//======================================^

^// c1c1 minp AND model^

^//======================================^

^// Creating step like function^

^//======================================^

^network DSignal(species X⁺ , function f) {^

^number precision = 1000^

^# ->{{precision*pos(f())}} X⁺; X⁺ ->{precision} #^

^}^

^//======================================^

^// Declaration of species^

^//======================================^

^species {A⁺,S,X1,X1a,X2,X2a,Y,Ya,Z,Za,K,PP,C1,C1a,C2a,C3}^

^//======================================^

^// Initial condition^

^//======================================^

^amount A⁺@ 0M^

^amount X1 @ 60 M^

^amount X1a @ 0 M^

^amount X2 @ 60 M^

^amount X2a @ 0 M^

^amount Y @ 60 M^

^amount Ya @ 0 M^

^amount Z @ 60 M^

^amount Za @ 0 M^

^amount S@ 0M^

^amount K@ 1M^

^amount PP@ 1M^

^amount C1@ 0M^

^amount C1a@ 0M^

^amount C2a@ 0M^

^amount C3@ 0M^

^//======================================^

^// Parameter value^

^//======================================^

^number k1 = 1^

^number k2 = 1^

^number k3 = 1^

^number kp = 10^

^number ka = 5^

^number kpp = 40^

^//======================================^

^// Input Generation^

^//======================================^

^DSignal(A⁺, fun(){cond(time<40,0.25,cond(time<80,1.5,cond(time<120,3,cond(time<160,2,0.5))))})^

^//======================================^

^// Plot input and output^

^//======================================^

^report S,Za^

^//======================================^

^//The mass-action kinetics reactions:^

^//======================================^

^A⁺ ->{200} S + S + A⁺ // stochastic input generation^

^S ->{200} # // self-degradation^

^X1 + K ->{kp} K + X1a // phosphorylation reaction, i.e. background reaction^

^X1 + S ->{ka} S + X1a // influence of stochastic input on the node of the motif^

^X1a + PP ->{kpp} PP + X1 // dephosphorylation reaction^

^X2 + K ->{kp} K + X2a // phosphorylation reaction, i.e. background reaction^

^X2 + S ->{ka} S + X2a // influence of stochastic input on the node of the motif^

^X2a + PP ->{kpp} PP + X2 // dephosphorylation reaction^

^Y + K ->{kp} K + Ya // phosphorylation reaction, i.e. background reaction^

^X1a + X2a {200} <->{200} C1 // C1 complex formation from X1a and X2a^

^C1 + PP ->{kpp} X1 + X2a + PP // dephosphorylation of C1 complex^

^C1 + PP ->{kpp} X1a + X2 + PP // dephosphorylation of C1 complex^

^Y + C1 ->{k1} C1 + Ya // activation of Y into Ya through AND connectivity^

^Ya + PP ->{kpp} PP + Y // dephosphorylation reaction^

^Z + K ->{kp} K + Za // phosphorylation reaction, i.e. background reaction^

^Ya + X1a {200} <->{200} C1a // C1a complex formation from X1a and Ya^

^C1a + PP ->{kpp} Y + X1a + PP // dephosphorylation of C1a complex^

^C1a + PP ->{kpp} Ya + X1 + PP // dephosphorylation of C1a complex^

^Ya + X2a {200} <->{200} C2a // C2a complex formation from X2a and Ya^

^C2a + PP ->{kpp} Y + X2a + PP // dephosphorylation of C2a complex^

^C2a + PP ->{kpp} Ya + X2 + PP // dephosphorylation of C2a complex^

^C1 + Ya {200} <->{200} C3 // C3 complex formation from C1 and Ya^

^C3 + PP ->{kpp} Y + C1 + PP // dephosphorylation of C3 complex^

^C1a + X2a {200} <->{200} C3 // C3 complex formation from C1a and X2a^

^C3 + PP ->{kpp} X2 + C1a + PP // dephosphorylation of C3 complex^

^C2a + X1a {200} <->{200} C3 // C3 complex formation from C2a and X1a^

^C3 + PP ->{kpp} X1 + C2a + PP // dephosphorylation of C3 complex^

^Z + C3 ->{k2} C3 + Za // activation of Z into Za through AND connectivity^

^Za + PP ->{kpp} PP + Z // dephosphorylation reaction^

^//======================================^

^// Total time of simulation^

^//======================================^

^equilibrate for 200^

^//======================================^

^// End^

^//======================================^

**Kaemika code for LNA computation of i2i2-mint-OR model under one step post translational modification:**

^//======================================^

^// i2i2 mint OR model^

^//======================================^

^// Creating step like function^

^//======================================^

^network DSignal(species X⁺ , function f) {^

^number precision = 1000^

^# ->{{precision*pos(f())}} X⁺; X⁺ ->{precision} #^

^}^

^//======================================^

^// Declaration of species^

^//======================================^

^species {A⁺,S,X,Xa,Y1,Y1a,Y2,Y2a,Z,Za,K,PP}^

^//======================================^

^// Initial condition^

^//======================================^

^amount A⁺@ 0M^

^amount X @ 60 M^

^amount Xa @ 0 M^

^amount Y1 @ 60 M^

^amount Y1a @ 0 M^

^amount Y2 @ 60 M^

^amount Y2a @ 0 M^

^amount Z @ 60 M^

^amount Za @ 0 M^

^amount S@ 0M^

^amount K@ 1M^

^amount PP@ 1M^

^//======================================^

^// Parameter value^

^//======================================^

^number k1 = 1^

^number k2 = 1^

^number k3 = 1^

^number kp = 10^

^number ka = 5^

^number kpp = 40^

^//======================================^

^// Input Generation^

^//======================================^

^DSignal(A⁺, fun(){cond(time<40,0.25,cond(time<80,1.5,cond(time<120,3,cond(time<160,2,0.5))))})^

^//======================================^

^// Plot input and output^

^//======================================^

^report S,Za^

^//======================================^

^//The mass-action kinetics reactions:^

^//======================================^

^A⁺ ->{200} S + S + A⁺ // stochastic input generation^

^S ->{200} # // self-degradation^

^X + K ->{kp} K + Xa // phosphorylation reaction, i.e. background reaction^

^X + S ->{ka} S + Xa // influence of stochastic input on the node of the motif^

^Xa + PP ->{kpp} PP + X // dephosphorylation reaction^

^Y1 + K ->{kp} K + Y1a // phosphorylation reaction, i.e. background reaction^

^Y1a + Xa ->{k1} Xa + Y1 // inhibition of Y1a to Y1^

^Y1a + PP ->{kpp} PP + Y1 // dephosphorylation reaction^

^Y2 + K ->{kp} K + Y2a // phosphorylation reaction, i.e. background reaction^

^Y2a + Xa ->{k1} Xa + Y2 // inhibition of Y2a to Y2^

^Y2a + PP ->{kpp} PP + Y2 // dephosphorylation reaction^

^Z + K ->{kp} K + Za // phosphorylation reaction, i.e. background reaction^

^Za + Y1a ->{k2} Y1a + Z // inhibition of Za into Z through OR connectivity^

^Za + Y2a ->{k2} Y2a + Z // inhibition of Za into Z through OR connectivity^

^Za + Xa ->{k3} Xa + Z // inhibition of Za into Z through OR connectivity^

^Za + PP ->{kpp} PP + Z // dephosphorylation reaction^

^//======================================^

^// Total time of simulation^

^//======================================^

^equilibrate for 200^

^//======================================^

^// End^

^//======================================^
