# Supplementary material for: Systematic analysis of noise reduction properties of coupled and isolated feed-forward loops
Source: PLoS Comput Biol. 2021 Dec 3;17(12):e1009622. doi: 10.1371/journal.pcbi.1009622 (PMC8641863; doi:10.1371/journal.pcbi.1009622)
Supplement: Attachment — Submitted filename: PCOMPBIOL-D-21-00869-report.pdf [file pcbi.1009622.s011.pdf]

Manuscript Number: PCOMPBIOL-D-21-00869
Title: Systematic Analysis of Noise Reduction Properties of Coupled and Isolated Feed-Forward Loops
Author(s): Suchana Chakravarty and Attila Csikasz-Nagy

The submitted manuscript presents a comprehensive study to address noise reduction and signal transmission capacity of coupled and isolated feed-forward loops. To this end, the authors propose a framework of theoretical model followed by extensive computational analysis. Although the authors tried to present their results in the best possible manner, I have certain queries which might improve the quality of the paper.

Major point:

1) I do not understand why the authors went for two-step modification models. As per Uri Alon's studies, classical feed-forward loops (FFLs) are mostly part of the gene regulatory network (GRN). Considering this one-step modification model is enough to analyze their mathematical model. Am I missing something here? If so, please put a brief discussion in the revised version.

2) The authors have used multiple levels of noisy input signals in their study. This means that the system never reaches a steady state and the authors mainly observe transient dynamics. In a time window of 200 min, they observe five different levels of Fano factor (FF). Which of these FF is optimum for a particular structure in noise reduction and enhanced signal transduction capacity?

Minor point:

1) The legends in the figures are too small.

2) Some of the sentences are too long.
