# Supplementary material for: Systematic analysis of noise reduction properties of coupled and isolated feed-forward loops
Source: PLoS Comput Biol. 2021 Dec 3;17(12):e1009622. doi: 10.1371/journal.pcbi.1009622 (PMC8641863; doi:10.1371/journal.pcbi.1009622)
Supplement: Attachment — Submitted filename: Review_v1.docx [file pcbi.1009622.s010.docx]

Overview

This paper systematically investigates various feed-forward loop (FFL) motifs and coupled systems and then identifies which FFL and coupled systems are good at noise reduction or signal transduction performances. The noise reduction and the signal transduction performances are quantified by the Fano factor and input-output relation slope, respectively. Based on these two measures, the authors conclude that coupling of two coherent 2 or one coherent 1 and one incoherent 4 FFLs (=what they listed in their discussion) can give the best performance. Moreover, the authors conclude that the OR-gates are better noise reducer than the AND-gates in general and nonlinear transition from multi-input steps make increase noise and signal transducing capabilities at the same time.

I like this work because this work would give clear guide for using FFLs motifs and their combination as control circuits. However, I have several concerns about the reliability of some conclusions. I know that a single paper cannot test all possible combinations, which may be infinitely many, of experiments. Thus, I don’t think that the authors need to perform all of combinations but still more analysis is needed at least for major conclusions. Please see below detail comments.

Major questions/comments

1. The Fano factor is sort of reference noise level for Poisson processes. However, it is not dimensionless (i.e., unit-less) quantity so it would differ when the absolute level of input data changes. Thus, I wonder how can the Fano factor be a 'fair' measure of the noise reduction.

2. I have concern with the parameter choices. Are the results universal? Are the conclusions made by the authors still valid with other choice of parameters? 'Systematic' analysis should contain not only the various motifs and systems but also parameter universality.

3. In the text above Figure 5, there is sentences: “On the same plots we can notice that coupled FFLs with AND gates tend to have a worse noise reducing capability than networks with OR gates (Fig. 5). On the busier figure 3 it was harder to notice this trend, but the same relation holds for all networks.” Because this is one of the authors’ main conclusions it would be great to provide a figure that clearly illustrates this relationship, e.g., box plots for the differences between the Fano factors from each AND-gate and OR-gate; x-axis for the Fano factors from AND-gates and y-axis for the Fano factors from OR-gates.

4. The authors claim that nonlinear transitions from multi-input steps of the pathways leads to noisier signal and increase of signal transducing capabilities. However, it is based on the results from isolated FFLs c1 and coupled FFLs c1c1 while there are still many other combinations of FFLs. How can we guarantee that this result would be preserved for other combinations of FFLs?

5. The authors have to fix either figures or texts. Specifically, in Figure 2D, the authors said that they picked input = 3 Fano factors, but it is not true; they are the Fano factors for input = 6. They have to match. Since the Fano factors are shown also in Figures 3, 5, and S5, author may have to fix these figures as well (I am not sure because the calibrations are not clear). This editing may not affect the major conclusion of this work (e.g., the best combination of motifs). However, if it does, then the authors have to adjust their conclusion accordingly.

Minor questions/comments

1. The first paragraph in Introduction is too long and contains multiple contexts. Please divide to several paragraphs.

2. In the first paragraph in Introduction, I recommend to refer corresponding Supplementary figures when the authors introduce various FFLs. It would improve the readability.

3. Why were the 'up-and-down' input signal used? It appears that the input and output data are used in only their stationary state, not transient. In other words, the five different input level can be regarded as five independent simulations.

4. In Figure 3, there are too many colors to distinguish them. I recommend using color and symbol at the same time for the visibility. It is important because the authors claim the best performing noise reducer and signal transducer based on the results shown in Figure 3.

5. Please write the subpanel letter (A, B, C) at the top-left corner of each panel for the visibility.

6. In Figures 5 and S5, there is ‘-2s’ in the names of some motifs, which represents ‘two-step’ modification steps. However, there is no explicit designation of its meaning. Please provide the meaning of ‘-2s’ in the main text or figure legend.

7. Although they already provided the description in Supplementary Text 1 and 'Kaemika' tool code, it would be great to describe detail equation at least for one motif.
