# Supplementary material for: Systematic analysis of noise reduction properties of coupled and isolated feed-forward loops
Source: PLoS Comput Biol. 2021 Dec 3;17(12):e1009622. doi: 10.1371/journal.pcbi.1009622 (PMC8641863; doi:10.1371/journal.pcbi.1009622)
Supplement: Attachment — Submitted filename: Review_Answers.docx [file pcbi.1009622.s012.docx]

Manuscript Number: PCOMPBIOL-D-21-00869

Title: Systematic Analysis of Noise Reduction Properties of Coupled and Isolated Feed-Forward Loops

Author(s): Suchana Chakravarty and Attila Csikasz-Nagy

We appreciate the referees' constructive criticism. Their suggestions greatly aided us in improving the manuscript, updating the figures, and making it more readable.

**Reviewer 1:**

We are grateful to reviewer 1 for describing our research as "interesting" , “timely” and deeming it "well executed " and “well written”.

**Specific remarks:**

1.1 The Fano factor measures burstiness (how different a process is from the Poisson process). However, this is not the only measure of noise magnitude. To characterize the standard deviation in terms of the mean, the Coefficient of Variation (CV, defined as standard deviation/mean) is very widely used. The CV may be preferable for characterizing the quality of signal transduction, whereas the FF is most appropriate to characterize the burstiness of gene expression . For this reason, all the plots showing Fano Factors versus slope should also be made for the CV, using a constant CV at the input.

We agree with the referee that the Fano factor determines how distinct a process is from a Poisson process. We have chosen to report Fano values as these are less dependent on the mean value of noisy processes (all five steps of the super-Poissonian noisy input process shows the same Fano factor of 1.83. The Fano factor can be thought of as a noise-to-signal ratio; it is a measure of the reliability with which the random variable can be estimated from a time window that contains several random events on average. Still we appreciate the suggestion of the referee and agree that coefficient of variation might be a better measure of signal to noise ratio (or the reciprocal of thereof). Thus we have changed the noise measures on all our plots to %CV, and kept Fano only on Figure 1 to explain the difference between the two measures.

Below we show to the referee that qualitatively (as expected from the definitions) there is no major difference between plots of Fano or CV% (Fig. A1, Fig. A2, Fig. A3). Especially the order of the performance of the networks does not show any changes.

**Fig. A1: Panels A and B illustrate the noise reduction and signal transduction properties of isolated FFLs.** Panel A depicts the Fano factor as a noise measurement, whereas panel B depicts the noise measurement as a percentage of coefficient of variation (%CV). All simulations were carried out using one-step post-translational modification models and the parameter values described in Supplementary Texts S1.

**Fig. A2: The noise reduction and signal transduction characteristics of multi input coupled FFLs.** The Fano factor is depicted as a noise measurement in panel A, while the noise measurement is depicted as a percentage of the coefficient of variation in panel B (%CV). All simulations were run with one-step post-translational modification models and parameter values from Supplementary Text S1.

**Fig. A3:** **The noise reduction and signal transduction characteristics of multi intermediate coupled FFLs.** The Fano factor is depicted as a noise measurement in panel A, while the noise measurement is depicted as a percentage of the coefficient of variation in panel B (%CV). All simulations were run with one-step post-translational modification models and parameter values from Supplementary Text S1.

1.2 Keeping the Fano factor constant at the input makes the reader wonder, what would have happened at higher/lower input FF? It would be interesting to explore what happens if the input FF is higher or lower. The same question refers to the input CV.

We kept the Fano factor for the input at a fixed value to ensure the external noise on the system have a similar measure at all input levels. The question, the referee asks is relevant and we have run simulations with a higher input noise level (Fano = 2.33 instead of 1.83), while ensuring that the mean input values are the same as before. We plotted how %CV and slope of signal transduction measure has changed in the best and worst performing noise reducer networks. The plot below shows that the final noise in the output layer is rather independent on the level of input noise, as the difference in output noise is similar with different input noise levels (Fig. A4).

**Fig. A4: Variations in input noise levels have no effect on output noise levels.** Panel A shows the original noisy input, while panel C shows the input with increased noise levels (achieved by producing three copies of species S, instead of two during noisy input generation). F_n_ is the Fano factor at the n-th steady state, and CV_n_ is the percent coefficient of variation. The output corresponding to panel A and C are B and D respectively, note that mean values are equal in the two cases. Panel E,F,G represents the %CV Vs Slope plots for isolated FFL (E), multi-input-coupled FFL (F) and multi-intermediate-coupled FFL (G). For both the original input (circle shaped marker) and input with increased noise level (x shaped marker), we have shown how percent CV changes with slope. Instead of plotting all networks, for clarity we just show results of various combinations of good (c1, i4) and bad (c2, c3) noise reducing FFLs.

These results (and question 4.3 below by referee #4) led us to introduce supplementary Figure S7, where we show that the noise reducing capacity of the posttranslationally controlled FFLs are their inherent feature as the output noise of these motifs is generally independent of input noise and naturally show a low noise level. On Fig S7 we investigate how increased or decreased noise levels are reduced by FFLs and show that the final noise in the output level is largely independent on input noise level. We also show here that FFLs, which we claim to be ‘Bad noise reducer’ could be a good noise reducer, but not at the output layer.

1.3 The manuscript examines signaling (post-translational) interactions. Similar network structures exist in transcriptional regulatory networks. It would be important to discuss how the results would apply to transcriptional regulatory interactions. A relevant reference for FFL clustering may be PMID:15018656.

Thank you for pointing this out, we have cited the article.

1.4 "...better noise reduction (lower Fano) correlates with better signal transduction (larger slope)." More specifically, this is true only for slope> 0. However, for slope< 0, better noise reduction (lower Fano) seems to correlate with worse signal transduction. Best signal transduction for slope< 0 is near -1, where the FF is the highest. The same is probably true for coupled motifs.

We agree with the referee that for slope > 0, better noise reduction (lower Fano) correlates with better signal transduction (higher slope). This holds true for both coupled and isolated feed-forward loops. We did not draw a particular conclusion on the networks with slope < 0 because these networks produce inverted output signals in relation to the input signal. Still, we can claim that a large negative slope means a good (inverted) signal transduction. Specifically, FFLs containing C2 coherent, inverting FFLs show good inverted signal transduction and even their noise reducing capability is not the worse among FFLs. Now we explain this in the main text.

Besides the FFL, negative feedback can effectively reduce noise, as the authors note. In addition to ref. [13], this is also described in PMID:10850721 and PMID:19279212, which may be worth citing.

We appreciate the referee pointing out these two works to us. We expand our discussion on other noise reducing network motifs and cite these papers in the introduction.

**Reviewer 2:**

We are thankful to reviewer 2 for finding our paper as ’systematic’ and ‘clear guide for using FFLs motifs and their combination as control circuits.’

**Specific remarks:**

2.1 The Fano factor is sort of reference noise level for Poisson processes. However, it is not dimensionless (i.e., unit-less) quantity so it would differ when the absolute level of input data changes. Thus, I wonder how can the Fano factor be a 'fair' measure of the noise reduction.

We appreciate the point made by the reviewer 2. Similar issues were raised by referee 1, so we have addressed this question under section 1.1.

2.2 I have concern with the parameter choices. Are the results universal? Are the conclusions made by the authors still valid with other choice of parameters? 'Systematic' analysis should contain not only the various motifs and systems but also parameter universality.

Certainly we did not want to claim we have tested all parameter combinations, but we wanted to limit ourselves to parameter choices where none of the simulated networks produce artificial behavior. We wanted to be especially sure that none of the FFL layers got irresponsive by moving all molecules into the activated or inactivated form. The total abundance of each species was set to 60 a.u., thus we wanted to keep all species during our 5 step input change simulations between 5 and 55 a.u. Still we had some flexibility in the signal processing speed of the networks and we have tested the model behaviors with 5 times slower rate than in the baseline model (k_1_=k_2_=k_3_=0.2, instead of 1). We report on the figure below the results of these simulations. These can be compared to figures 2D, 3 A, B of the main text. We claim that there is no qualitative difference between the plots.

**Fig A5.: Noise reducing capabilities at slower signal processing.** At input = 6 a.u., the Fano factors of each combined FFL were calculated and plotted against the slopes of the input-output relationships to show how well each multi-input coupled FFL (A), multi-intermediate FFL (B), and isolated FFL (C) network performs in noise reduction and signal transduction. These are similar plots to Fig. 2D, 3 A, B respectively, just with different signal transducing rates. All simulations were carried out using one-step post-translational modification models, with the following parameter sets: k_1_=k_2_=k_3_=0.2, k_p_=10, k_pp_=40, and k_a_=5.

2.3 In the text above Figure 5, there is sentences: “On the same plots we can notice that coupled FFLs with AND gates tend to have a worse noise reducing capability than networks with OR gates (Fig. 5). On the busier figure 3 it was harder to notice this trend, but the same relation holds for all networks.” Because this is one of the authors’ main conclusions it would be great to provide a figure that clearly illustrates this relationship, e.g., box plots for the differences between the Fano factors from each AND-gate and OR-gate; x-axis for the Fano factors from AND-gates and y-axis for the Fano factors from OR-gates.

Thank you for this suggestion. We added a new figure (Fig. 6) in the main manuscript where we show that good noise reducing FFLs always show a better noise reduction in the OR settings than in the AND settings. We have plotted all combinations there where both OR and AND gates can be considered.

2.4 The authors claim that nonlinear transitions from multi-input steps of the pathways leads to noisier signal and increase of signal transducing capabilities. However, it is based on the results from isolated FFLs c1 and coupled FFLs c1c1 while there are still many other combinations of FFLs. How can we guarantee that this result would be preserved for other combinations of FFLs?

To address this issue, Figure 5 and Figure S5 have been updated. Now we show the noise reducing and signal transducing capabilities of all the 75 networks in one step and two steps post-translational modifications. Similar color, but different shape labels of the 1-step and 2 step networks show that the trend of better noise reduction, but worse signal transduction in one-step networks holds in most models and where this is not true, the two values are quite similar.

2.5 The authors have to fix either figures or texts. Specifically, in Figure 2D, the authors said that they picked input = 3 Fano factors, but it is not true; they are the Fano factors for input = 6. They have to match. Since the Fano factors are shown also in Figures 3, 5, and S5, author may have to fix these figures as well (I am not sure because the calibrations are not clear). This editing may not affect the major conclusion of this work (e.g., the best combination of motifs). However, if it does, then the authors have to adjust their conclusion accordingly.

We would like to thank the referee for pointing out this type. We have picked input = 6, the value 3 was there by mistake.

**Minor remarks:**

2.6 The first paragraph in Introduction is too long and contains multiple contexts. Please divide to several paragraphs.

We have taken care of that.

2.7 In the first paragraph in Introduction, I recommend to refer corresponding Supplementary figures when the authors introduce various FFLs. It would improve the readability.

Done.

2.8 Why were the 'up-and-down' input signal used? It appears that the input and output data are used in only their stationary state, not transient. In other words, the five different input level can be regarded as five independent simulations.

Yes, we could have used five independent simulations instead 5 different input levels. However, we wanted to see how much delay we see for such step-like changes. Eventually we did not see much difference in these, which finding we now mention in the text.

2.9 In Figure 3, there are too many colors to distinguish them. I recommend using color and symbol at the same time for the visibility. It is important because the authors claim the best performing noise reducer and signal transducer based on the results shown in Figure 3.

There are 59 different combinations to be plotted, we have changed the labelling of figure 3, which we believe now gives a better explanation which point corresponds to which model.

2.10 Please write the subpanel letter (A, B, C) at the top-left corner of each panel for the visibility.

Some of the figures does not have empty space at the top-left corner. Where we could not squeeze the letters in we have moved them to the most appropriate location.

2.11 In Figures 5 and S5, there is ‘-2s’ in the names of some motifs, which represents ‘two-step’ modification steps. However, there is no explicit designation of its meaning. Please provide the meaning of ‘-2s’ in the main text or figure legend.

To prevent complication, we deleted the ‘-2s' from the legend and indicated clearly two steps modification on the image.

2.12 Although they already provided the description in Supplementary Text 1 and 'Kaemika' tool code, it would be great to describe detail equation at least for one motif.

Kaemika tool (Cardelli, Luca. "Kaemika app: Integrating protocols and chemical simulation." In International Conference on Computational Methods in Systems Biology, pp. 373-379. Springer, Cham, 2020.) requires as input all the chemical reactions considered and gives the mean values and with the linear noise approximation it also gives the estimated noise of the results. The methodology for this was published earlier (Cardelli, L., Kwiatkowska, M., & Laurenti, L. (2016). Stochastic analysis of chemical reaction networks using linear noise approximation. Biosystems, 149, 26-33.). Thus, no differential equations were written for the models. For clarity we have added the list of chemical reactions to the supplement.

**Reviewer 3:**

We are thankful to reviewer 3 for finding our computational analysis ‘extensive’.

**Specific remarks:**

3.1 I do not understand why the authors went for two-step modification models. As per Uri Alon’s studies, classical feed-forward loops (FFLs) are mostly part of the gene regulatory network (GRN). Considering this one-step modification model is enough to analyze their mathematical model. Am I missing something here? If so, please put a brief discussion in the revised version.

Indeed, most work so far on FFLs focused on gene regulatory networks. Here we rather focus on networks where interactions are based on posttranslational modifications. A typical example, we discuss in details in the introduction and also refer back to in the discussion, is the MAP-kinase pathway, which is often modelled as a two-step modification (phosphorylation) system. We have made this statement more clear in the abstract and introduction.

3.2 The authors have used multiple levels of noisy input signals in their study. This means that the system never reaches a steady state and the authors mainly observe transient dynamics. In a time window of 200 min, they observe five different levels of Fano factor (FF). Which of these FF is optimum for a particular structure in noise reduction and enhanced signal transduction capacity?

The transit dynamics were not discussed in details, since the transitions between steady states happened in a far faster timescale than we have investigated the systems. Indeed, for the calculations of mean and STD values at each of the five steady states we have dropped the first and last 5 arbitrary time units (of the total of 40 arbitrary time units with an input value) after each input change to give enough time for the systems to settle in the new states. We were not really looking for an ideal FF at the different values, rather wanted to see how FF depends on the changes in mean input levels.

**Minor remarks:**

3.3 The legends in the figures are too small.

We have increased the legends where we could or used same color text instead of legend to increase font size.

3.4 Some of the sentences are too long.

We went through the text and eliminated long sentences.

**Reviewer 4:**

We are grateful to reviewer 4 for finding the concept of combining FFL in noise reduction investigation to be "interesting."

**Specific remarks:**

4.1 The paper lacks a clear materials & methods section, and this it remains unclear what they actually did. I think this would be essential for the paper.

Indeed, we did not put a separate Methods section in the main text, but we found the details of this too technical and to include too many tables and equations, so moved the whole methods section to the supplement of the paper and cited it in the appropriate locations of the main text. The main methodological concepts are presented in the first paragraphs of the results section, but we felt these details are indeed parts of the results, rather than pure methods, so we did not name this part separately as Methods. We hope the referee can accept our choice in handling this!

4.2 From what I understand, they calculate noise using LNA given one set of parameters, and it remains unclear if this result is dependent on the choice of parameters. I suppose that it is very much dependent on the time scales of each reaction, and thus other topologies might perform much better when different time scales are involved in the different layers.

We have received a similar question from referee 2. We have addressed this issue above in section 2.2.

4.3 The paper provides no intuition why noise reduction occurs. Maybe write down the LNA equations of the best performing networks, then it might be apparent why noise cancels or is reduced, and which parameters are important.

The linear noise approximation was directly performed by the Kaemika (Cardelli, Luca. "Kaemika app: Integrating protocols and chemical simulation." In International Conference on Computational Methods in Systems Biology, pp. 373-379. Springer, Cham, 2020.) based on the methods by (Cardelli, L., Kwiatkowska, M., & Laurenti, L. (2016). Thus we did not directly derive the LNA equations of the investigated systems. To understand the way posttranslational FFLs reduce noise we investigated how noise reduction happens at each layers of the FFLs and found that top noise reducer FFLs show a pattern where in each layer the noise is getting lower and lower, while in worse performing FFLs this trend is not observed. We have added a new figure (Fig. 7) and a separate paragraph in the discussion of the main text to explain these findings. We really thank the referee for highlighting this point, which helped us a lot to improve our manuscript.

4.4 It is unclear if the Fano factor is not a good measure of how input noise is reduced, I would argue that the signal/noise ratio is much more important (imagine a network with a lot of input noise, i.e. where only the input noise is important - then the Fano factor would depend on the number of molecules of the output, whereas signal/noise would not). It would be therefore important for the authors to (i) clearly describe the Fano factor and the rational behind using it here.

We have addressed this in the section 1.1.

**Minor remarks:**

4.5 The paper needs a thorough proof-read, a re-edit of the figures (fonts very small). The authors should introduce the notation for the FFL-classes more clearly (maybe in an initial figure), so it is immediately clear what e.g. c1i3 is.

The manuscript was proof read now and we have also increased font sizes on figures. The detailed structure and details on all models were presented in the supplement (supplementary figure 1). We are clearly referring to this, when introducing the notation of FFL classes.
